# Congenital Cytomegalovirus Infection: A Narrative Review of the Issues in Screening and Management From a Panel of European Experts

**DOI:** 10.3389/fped.2020.00013

**Published:** 2020-01-31

**Authors:** Tiziana Lazzarotto, Daniel Blázquez-Gamero, Marie-Luce Delforge, Ina Foulon, Suzanne Luck, Susanne Modrow, Marianne Leruez-Ville

**Affiliations:** ^1^Virology Lab, Polyclinic St. Orsola Malpighi, University of Bologna, Bologna, Italy; ^2^Pediatric Infectious Diseases Unit, Hospital Universitario 12 de Octubre, Universidad Complutense, Instituto de Investigación Hospital 12 de Octubre (Imas12), Translational Research Network in Pediatric Infectious Diseases (RITIP), Madrid, Spain; ^3^CUB-Hôpital Erasme, Université Libre Bruxelles, Brussels, Belgium; ^4^Department of Otolaryngology - Head and Neck Surgery, Vrije Universiteit Brussel, Brussels, Belgium; ^5^Kingston Hospital NHS Trust, Kingston upon Thames, United Kingdom; ^6^Paediatric Infectious Diseases Research Group, St George's University of London, London, United Kingdom; ^7^Institute of Medical Microbiology, University of Regensburg, Regensburg, Germany; ^8^Hôpital Necker-Enfants Malades and Université Paris Descartes, Paris, France

**Keywords:** cytomegalovirus, neonatal screening, prenatal diagnosis, clinical laboratory techniques, congenital CMV, pregnancy, maternal screening

## Abstract

Maternal primary and non-primary cytomegalovirus (CMV) infection during pregnancy can result in *in utero* transmission to the developing fetus. Congenital CMV (cCMV) can result in significant morbidity, mortality or long-term sequelae, including sensorineural hearing loss, the most common sequela. As a leading cause of congenital infections worldwide, cCMV infection meets many of the criteria for screening. However, currently there are no universal programs that offer maternal or neonatal screening to identify infected mothers and infants, no vaccines to prevent infection, and no efficacious and safe therapies available for the treatment of maternal or fetal CMV infection. Data has shown that there are several maternal and neonatal screening strategies, and diagnostic methodologies, that allow the identification of those at risk of developing sequelae and adequately detect cCMV. Nevertheless, many questions remain unanswered in this field. Well-designed clinical trials to address several facets of CMV treatment (in pregnant women, CMV-infected fetuses and both symptomatic and asymptomatic neonates and children) are required. Prevention (vaccines), biology and transmission factors associated with non-primary CMV, and the cost-effectiveness of universal screening, all demand further exploration to fully realize the ultimate goal of preventing cCMV. In the meantime, prevention of primary infection during pregnancy should be championed to all by means of hygiene education.

## Introduction

Maternal primary and non-primary infection (exogenous reinfection with a different strain or endogenous viral reactivation) of cytomegalovirus (CMV) during pregnancy can result in *in utero* transmission to the fetus (1). Infants can be categorized as symptomatic or asymptomatic based on clinical symptoms/signs (Table 1) (2). Approximately 11% of live-born infants born with congenital CMV (cCMV) have abnormal clinical findings at birth (symptomatic) (3). Infants can experience substantial morbidity, mortality, and long-term sequelae, including sensorineural hearing loss (SNHL), the most common sequela (4, 5). Infants without symptoms at birth are also reported to be at risk of developing long-term hearing loss (6). As a leading cause of congenital infections worldwide (7), cCMV infection meets many of the criteria for screening: it is clinically important, well defined and prevalent (4). Nevertheless, neither universal antenatal screening for CMV during pregnancy nor universal neonatal screening is routinely recommended (8) and there remain several challenges that impede their implementation. Roche Centralised and Point of Care Solutions and Roche Molecular Diagnostics convened a group of CMV experts (microbiologists, virologists, and clinicians) to discuss and offer strategies to address these barriers and knowledge gaps. This paper provides an overview of those discussions and is a narrative review of serologic and viral nucleic acid screening and diagnostics in the context of maternal, fetal and neonatal CMV infection.

**Table 1:** Possible signs and symptoms in children with congenital CMV (reproduced from Luck et al.).

**CLINICALLY DETECTABLE SYMPTOMS/SIGNS**
**Physical examination**
Small for gestational age (birth weight < −2 standard deviations for gestational age) Microcephaly (head circumference < −2 standard deviations for gestational age) Petechiae or purpura (usually found within hours of birth and persist for several weeks) Blueberry muffin rash (intra dermal hematopoiesis) Jaundice^a^ Hepatomegaly Splenomegaly
**Neurologic physical examination**
Microcephaly (head circumference < −2 standard deviations for gestational age) Neurologic signs (lethargy, hypotonia, seizures, poor sucking reflex)
**ABNORMALITIES DETECTED INCIDENTALLY OR THROUGH**
**SUBSEQUENT INVESTIGATION/SPECIALIST EXAMINATION**
**Laboratory results**
Anemia Thrombocytopenia (occurs in the first week but platelets often increase spontaneously after the second week) Leukopenia, isolated neutropenia Elevated liver enzymes (alanine aminotransferase/aspartate aminotransferase) Conjugated hyperbilirubinemia
**Cerebrospinal fluid**
Abnormal cerebral fluid indices, positive CMV DNA
**Neuroimaging**
Calcifications, periventricular cysts, ventricular dilatation, subependymal pseudocysts, germinolytic cysts, white matter abnormalities, cortical atrophy, migration disorders, cerebellar hypoplasia, lenticulostriatal vasculopathy
**Hearing test**
Sensorineural hearing loss uni- or bilaterally
**Visual examination**
Chorioretinitis, retinal hemorrhage, optic atrophy, strabismus, cataracts

*CMV, cytomegalovirus*.

a *CMV-associated jaundice can be present at the first day after birth and usually persists longer than physiologic jaundice*.

## Maternal CMV Screening

CMV screening is offered to some pregnant women in parts of Europe, Israel, Australia and the USA in the setting of population-based studies, and independently of nationally endorsed screening programs (9). However, universal antenatal screening for CMV is not routinely recommended (8). Reasons not to screen include the absence of medication to prevent transmission and the difficulty of predicting sequelae (10).

The introduction of routine testing for CMV in pregnant women has several implications. Despite the difficulties mentioned above, the most important benefit of screening would be to identify fetuses at risk of developing sequelae.

Maternal screening, ideally early in the first trimester, would also identify those who were CMV-seronegative and thus allow information to be provided regarding hygiene and behavioral measures to prevent CMV infection. Evidence has shown that intervention based on the identification and hygiene counseling of CMV-seronegative pregnant women significantly prevents maternal infection (11). Hygiene counseling may also provide (as yet unproven) benefits for those who are seropositive.

During early pregnancy, repeat serologic screening with CMV-specific immunoglobulin G (IgG) and -M (IgM) antibodies of previously seronegative pregnant women at the end of the first trimester (or until week 20) would identify maternal primary CMV infection. Although there are no universally accepted guidelines, testing before 18–20 weeks of pregnancy is reasonable in order to identify late seroconversion at the end of the first trimester and implement fetal investigations. In the event of seroconversion, parents should be informed of the risk of vertical transmission [32% (3)] and the possible consequences.

Other strategies that could be put in place include maternal screening at the first prenatal visit and at birth, and neonatal screening at birth for those whose mothers tested seropositive, with diagnosis of the neonate by saliva or urine CMV DNA detection. Previously, this approach to targeted testing detected 82% of all cCMV infections (12). Notably, this study utilized culture for diagnosis; the detection rate is likely to be improved with polymerase chain reaction (PCR). This approach may be more cost-effective than screening all children.

However, screening tests do not identify which mothers will transmit the virus (13). Moreover, there are no surrogate markers to predict whether infection in the infant will lead to long-term sequelae.

Lastly, there are also risks for the mother associated with maternal screening for CMV that include the stress of having extra tests, the potential for unnecessary terminations (14), the potential risk of miscarriage or stillbirth from confirmatory amniocentesis [inversely correlated to skill/experience of operator (15)], and the cost. The challenge is in providing women with choice and information in the context of population-based economics.

### Serologic and Molecular Testing for Maternal CMV Infection

Primary infections can be identified by serologic testing. During pregnancy, IgG and IgM serology is the preferred option; IgG avidity testing should be used only if CMV-specific IgM antibodies are positive. Many laboratories consider IgM-positive results, in combination with IgG avidity results, to discriminate between primary and non-primary CMV infections (16). Low CMV IgG avidity indicates primary infection within the preceding 3–4 months, with an increased risk of intrauterine transmission to the fetus (17).

CMV-specific serology testing is most useful in the first trimester due to the increased severity of disease when primary infection occurs during the embryonic or early fetal period (18). Quantitative IgG testing is helpful to detect seroconversion and the stage of infection. Low levels of CMV-IgG antibodies in maternal serum samples present challenges for the clinician: low IgG levels can be associated with both a true positive or a false-positive result (19), so clear guidelines are needed for the appropriate interpretation of serology results.

All serologic kits vary considerably in their accuracy of the “low” range of IgG values; a very low value in one test may be negative using a different test. CMV-IgG avidity testing should not be performed on serum samples with low IgG levels as these can give inappropriate IgG avidity results (20). An incorrect classification of primary CMV infection can lead to inappropriate management. Finally, maternal serology screening can be falsely reassuring as non-primary maternal infections will not be recognized: in Europe, this represents around 50% of all cCMV cases (21, 22).

The new WHO standard (23) was established for the calibration of anti-CMV IgG diagnostic kits with quantitative test interpretation and as an aid in the interpretation of serologic results in the framework of different assays, platforms, and clinical settings. The CMV standard could be of value, although in other settings such as rubella or toxoplasmosis screening, the use of a standard for calibrating IgG assays has proven to be suboptimal (24, 25). An algorithm for dealing with low positive IgG samples may be more useful than a WHO standard. In particular, in the absence of a gold standard method, an equivocal IgG serologic assay result in a pregnant woman should be considered negative. This strategy will ensure that these women are assigned to the highest CMV risk group for pregnancy outcome.

The value of viral DNA detection and quantification in blood, saliva, or urine to help determine the timing of maternal infection, or to estimate the risk of fetal transmission, is not yet established. Notably, two studies have demonstrated that persisting levels of maternal DNAemia during primary CMV infection at the moment of amniocentesis correlate with a high risk of CMV transmission to the fetus (26, 27), whilst one other study has shown that the presence of CMV DNA in maternal urine and maternal blood correlated with transmission of CMV to offspring (28).

### Prenatal Diagnosis of Fetal CMV Infection

Ultrasound imaging has poor sensitivity in diagnosing fetal CMV infection (29) but is a useful tool to predict the prognosis of fetal infection. Diagnosis of fetal CMV infection by CMV PCR in the amniotic fluid can be made with high sensitivity and specificity by amniocentesis after 20–21 weeks' gestation (30) [and >8 weeks after estimated maternal seroconversion (31)] and is the best available prenatal diagnostic tool (32). When the diagnosis of fetal infection is by way of amniocentesis, the prognostic evaluation of fetal infection relies on imaging using a combination of ultrasound and cerebral magnetic resonance imaging (MRI). Several studies have identified a residual risk of hearing loss at birth when imaging (ultrasound and/or MRI) examination was considered to be normal (33–38).

## Neonatal CMV Screening

Neonatal CMV screening would enable early detection of cCMV (following primary and non-primary maternal infection), but universal neonatal screening for CMV is currently not recommended by any public health body. Data from Uematsu and colleagues emphasize that without neonatal screening some infected neonates that develop neurological sequelae may go unrecognized (39).

Although universal screening is not performed, targeted screening of newborns who fail the neonatal hearing test has been implemented in some hospitals and states in the USA (40, 41). In the UK, Belgium and Australia, targeted testing of infants who were referred for further audiological testing (after failing the routine hearing screening) has also been trialed with some success (42–44). This combination of targeted newborn screening and early detection and interventions is likely to benefit children with cCMV (45).

Additionally, the costs associated with targeted neonatal screening look favorable compared with other screening programs (46, 47). However, this targeted approach would miss those CMV-positive infants who pass the newborn hearing test but are still at risk for late-onset SNHL (40, 48). In one study, 43% of infants with CMV-related SNHL in the neonatal period and cCMV infants who are at risk for late-onset SNHL were not identified by newborn hearing screening (49).

There are risks associated with neonatal screening, such as the potential for parental anxiety while waiting for confirmatory testing results. In addition, there may be anxiety related to the extended period of audiological monitoring that a cCMV-positive infant must undergo [up to 6 years (2, 50, 51)]. Most cCMV infections are asymptomatic and do not present a risk for the onset of late sequelae (5). Recent data demonstrated that primary maternal infections before the 14th week of pregnancy, the presence of a disseminated infection at birth, and imaging abnormalities in the neonate were risk factors for SNHL (52). This is a step toward the development of neonatal predictive markers that can be used to identify those at high risk of developing sequelae.

### Diagnosis and Screening of Neonatal Infection

Testing is recommended for those who have any condition that might be indicative of intrauterine CMV infection (2). Traditionally, viral isolation and culture from urine or saliva was the standard for diagnosing cCMV infection (53). Since PCR exhibits high sensitivity (54, 55), this is now the preferred option. False-positive tests have been reported for saliva, and therefore any positive saliva result should be confirmed by CMV detection in urine (21).

It should be noted that if the diagnosis is made after the first 2–3 weeks of life (2, 9), infection may have been postnatally acquired and attributable to infected breast milk from a seropositive mother (56), rather than cCMV. In this instance, congenital infection must be confirmed by detection of CMV from a sample taken at birth.

Saliva can be used to screen for cCMV; however, as this specimen type is not routinely collected from neonates, a change in infrastructure would be required before this could be rolled out on a large scale. As such, alternative technologies for universal screening are currently under evaluation. Due to widespread utilization in neonatal screening for other conditions, there has been much interest in using dried blood spots (DBS) taken at birth for CMV screening. However, screening DBS is less sensitive than PCR testing of saliva, with sensitivity ranging between 28 and 100% (57), and is contingent upon the method of extraction and DNA amplification and the patient group selected. The recent standardization of viral DNA extraction and innovative PCR techniques has led to improved sensitivity of DBS screening to around 80% (58). A potential limitation in the use of DBS is that only 80–90% of congenitally infected infants have detectable CMV in their blood soon after birth (59, 60). Despite this, the sensitivity of DBS screening has been shown to adequately detect those most at risk of developing SNHL (61).

Stored DBS can be used to diagnose cCMV retrospectively (2). In some countries (e.g., Germany), the use of DBS for retrospective diagnosis or screening of newborns is hampered by the destruction of samples after 3 months (62) due to data protection requirements. Thus, for certain countries, regulatory changes may be necessary to allow long-term storage and use in this context.

## What are the Gaps in our Understanding?

Although significant advancements have been made, many questions remain unanswered in this field (Table 2). Well-designed clinical trials to address several facets of CMV treatment (in pregnant women, CMV-infected fetuses and both symptomatic and asymptomatic neonates and children) are required. Prevention (vaccines), biology, and transmission factors associated with non-primary CMV, and the cost-effectiveness of universal screening, all demand further exploration to fully realize the ultimate goal of preventing cCMV.

**Table 2:** Studies required to improve the understanding of congenital CMV.

**DIAGNOSIS**	
Non-primary infection in pregnant women	Identify virologic and immunological markers predictive of cCMV in women seropositive before pregnancy Identify virologic and immunological CMV-specific tests to properly diagnose maternal non-primary infection in pregnancy
Universal neonatal screening	Evaluate the performance of CMV PCR in DBS to identify neonates with sequelae and determine cost-effectiveness
**PREVENTION**	
Prevention of fetal transmission in maternal primary infection	Randomized controlled studies with new antiviral drugs
Prevention of maternal infection	Randomized controlled studies of optimal education methods and efficacy of hygiene measures in general population
**TREATMENT**	
Treatment of fetal infection	Randomized controlled studies with new available antiviral drugs
Treatment of infection in the neonate	Randomized controlled studies: – with new antiviral drugs; – with known antiviral drugs to confirm the effectiveness of known therapies; – in asymptomatic babies with no clinical examination abnormalities at birth (including those developing later hearing loss); – to determine the optimal duration and dosage of treatment Registries of long-term treatment sequelae

*(c)CMV, (congenital) cytomegalovirus; DBS, dried blood spot; PCR, polymerase chain reaction*.

Currently, treatment with immunoglobulins or antiviral therapy to prevent intrauterine transmission of CMV in pregnant women with primary CMV infection is not recommended as studies have not yet conclusively shown a benefit (63–66). Data from a non-randomized study showed that biweekly administration of hyperimmunoglobulin until 20 weeks' gestation successfully prevented maternal-fetal transmission of primary infections (65). These data need to be confirmed by a randomized clinical trial (67); if the results are confirmed, two-weekly intervals for testing seronegative women would be necessary. It has been suggested that a study that demonstrates treatment efficacy resulting in at least a 47% reduction in cCMV disease would make universal screening and treating for primary CMV in pregnancy cost-effective (10).

Whilst recent improvements in screening and diagnosis allow detection of primary CMV infection in pregnancy, unfortunately treatment options for CMV-infected fetuses and neonates are limited due to insufficient evidence for safety and effectiveness. Consequently, routine antiviral therapy to treat fetal CMV during pregnancy is not recommended (9). In infants with clinical disease at birth, early intervention with ganciclovir or valganciclovir can prevent hearing deterioration and improve developmental outcomes, although both treatments are associated with neutropenia (68, 69) and other possible long-term effects (70). Currently, valganciclovir treatment is recommended based on severity or number of symptoms. A European expert consensus statement recommends that treatment with oral valganciclovir (intravenous ganciclovir under certain circumstances) is only for those with: evidence of central nervous system disease; evidence of life-threatening disease, severe single-organ disease or multi-organ involvement; “moderate” cCMV disease once discussed on a case-by-case basis with a clinician with experience of managing infants with cCMV (2). The informal International Congenital Cytomegalovirus Recommendations Group recommend oral valganciclovir treatment for those neonates with “moderately” to “severely” symptomatic cCMV disease (9). Further development of efficacious antivirals with an acceptable safety profile is required.

Letermovir is a new agent approved for use in the prophylaxis of CMV infection in CMV-seropositive recipients of an allogeneic hematopoietic stem cell transplant over the age of 18 years (71). A recent case study revealed potential efficacy in pediatric allogeneic hematopoietic stem cell transplant patients (72). Further studies are required to determine whether it is safe and effective for treating those with cCMV.

More asymptomatic infants will be discovered if screening programs become widespread. Currently, antiviral therapy for asymptomatic infants is not recommended (2) but, like symptomatic infants, they are at risk of developing late-onset sequelae (53). Trials that investigate which interventions are effective and safe for asymptomatic infants or those with isolated hearing loss or subtle neuroimaging abnormalities, as well as older children that develop late-onset hearing loss, are necessary. Since clinical trials are ongoing (73–75), some of these points will hopefully be clarified in the next few years and the best management plan for this population determined.

As well as trials targeting treatment for specific populations, data on prevention are also required. It is known that natural infection confers some protection against both horizontal and vertical transmission (13, 76), therefore the development of an effective vaccine is feasible. Clinical trials of CMV vaccines should evaluate protection against cCMV infection (77). Recent modeling data of a single fictional cohort of 390,000 adolescent women suggest that vaccination could be cost-effective (78).

In the absence of a vaccine to prevent infection, a greater focus on education and prevention strategies for cCMV infection are needed for women intending to become pregnant, those already pregnant, and healthcare professionals alike. Preconception screening in those attending a fertility clinic, with resultant counseling to improve personal hygiene in those who were not immune to CMV, has shown that hygiene counseling (albeit in this highly selected cohort) is effective in reducing CMV exposure (79). In pregnant women, hygiene counseling of CMV-seronegative pregnant women significantly prevents maternal infection (11). De Vries and colleagues showed that non-primary infections account for the majority of CMV-related hearing loss, suggesting prevention research should encompass all pregnant women, not just those who are seronegative (80). Therefore, the current method aimed at preventing transmission of CMV—education concerning hygiene measures to be taken around small children [such as avoiding kissing babies on the mouth, not sharing cutlery with young children, and hand hygiene after a diaper change (11)]—should be performed regardless of knowing the mother's serostatus. However, in the general population, there is inadequate evidence to show that education translates into a decrease in maternal infection (4). Studies assessing the efficacy of hygiene measures on the prevention of CMV infection in pregnancy, and resultant cCMV, are necessary in women of reproductive age and are ongoing in the UK (81).

Clinicians do not know whether a non-primary infection is a reactivation or an infection with another strain of CMV. This distinction may be important in understanding the etiology of CMV disease, thus more research on the role of non-primary maternal CMV infections in congenital infection is necessary. At present, there are no tools validated to identify women at risk of transmitting the virus after a non-primary infection.

Finally, further evidence of cost-effectiveness is required. Whilst the cost-effectiveness of universal and targeted newborn cCMV screening programs has been assessed in the UK and USA (46, 47), and the economic burden of cCMV in the UK estimated (82), currently there are insufficient cost-benefit data, which hinders the implementation of screening.

## Conclusions

cCMV infection results in significant consequences for the infected neonate. Despite this, universal maternal or neonatal screening for CMV and cCMV is not routinely recommended. A summary of our current recommendations for diagnosis, screening, and prevention is provided in Table 3. Presently there are significant gaps in understanding that prevent the implementation of universal screening, including insufficient data on cost-effectiveness and the lack of evidence for safe and efficacious treatments for those infected. Additionally, further data on non-primary maternal infection and the risk of cCMV infection are necessary. In the near future, we are confident that many aspects related to diagnosis, maternal and fetal therapy, and active prevention will surely present an improvement and our recommendations may change. Until then, and in the absence of a vaccine, hygiene recommendations to prevent CMV infection should be made to all pregnant women.

**Table 3:** Expert panel recommendations for the diagnosis, screening, and prevention of CMV infection.

**RECOMMENDATIONS FOR DIAGNOSIS**	
Primary infection in pregnant women	IgG, IgM, IgG avidity if positive IgM and IgG
Non-primary infection in pregnant women	No tools validated
Fetal infection	CMV PCR in amniotic fluid after 20 weeks and more than 8 weeks after presumed onset of maternal primary infection
Neonatal infection	CMV PCR in saliva or urine collected in the first 3 weeks of life
Retrospective diagnosis in toddlers with compatible symptoms	CMV PCR in neonatal DBS
**RECOMMENDATIONS FOR SCREENING AND PREVENTION**	
Primary prevention of maternal primary infection	Information for pregnant women on cCMV and application of hygienic measures to prevent maternal infection
Infection in pregnant women	No recommendation for screening Information for pregnant women on cCMV and application of hygienic measures to prevent maternal infection
Universal neonatal screening	No recommendation
Targeted testing in neonates who failed universal hearing screening	CMV PCR in saliva (if positive, confirm in urine or by DBS PCR if the infant is > 3 weeks of age)

*(c)CMV, (congenital) cytomegalovirus; DBS, dried blood spot; Ig, immunoglobulin; PCR, polymerase chain reaction*.

## Author Contributions

TL and ML-V wrote the first draft of the manuscript. All authors (TL, DB-G, M-LD, IF, SL, SM, and ML-V) contributed to manuscript revision, read and approved the submitted version.

### Conflict of Interest

TL, M-LD, IF, SL, and SM report personal fees from Roche Diagnostics Ltd. DB-G reports personal fees from Roche Diagnostics Ltd. and MSD. ML-V reports personal fees from Roche Diagnostics Ltd., non-financial and other support from BioMérieux, non-financial support from Abbott, and non-financial support from Ferring SAS.

## Abbreviations

cCMV: Congenital cytomegalovirus
CMV: Cytomegalovirus
DBS: dried blood spots
IgG: Immunoglobulin G
IgM: Immunoglobulin M
MRI: magnetic resonance imaging
PCR: polymerase chain reaction
SNHL: Sensorineural hearing loss.

## References

[1] Cannon MJ Schmid DS Hyde TB. Review of cytomegalovirus seroprevalence and demographic characteristics associated with infection. Rev Med Virol. (2010) 20:202–13. 10.1002/rmv.655 20564615

[2] Luck SE Wieringa JW Blázquez-Gamero D Henneke P Schuster K Butler K. Congenital cytomegalovirus: a European expert consensus statement on diagnosis and management. Pediatr Infect Dis J. (2017) 36:1205–13. 10.1097/INF.0000000000001763 29140947

[3] Kenneson A Cannon MJ. Review and meta-analysis of the epidemiology of congenital cytomegalovirus (CMV) infection. Rev Med Virol. (2007) 17:253–76. 10.1002/rmv.535 17579921

[4] Johnson JM Anderson BL. Cytomegalovirus: should we screen pregnant women for primary infection? Am J Perinatol. (2013) 30:121–4. 10.1055/s-0032-1333133 23292913

[5] Goderis J De Leenheer E Smets K Van Hoecke H Keymeulen A Dhooge I. Hearing loss and congenital CMV infection: a systematic review. Pediatrics. (2014) 134:972–82. 10.1542/peds.2014-1173 25349318

[6] Bartlett AW McMullan B Rawlinson WD Palasanthiran P Hearing and neurodevelopmental outcomes for children with asymptomatic congenital cytomegalovirus infection: a systematic review. Rev Med Virol. (2017) 27:e1938 10.1002/rmv.1938 28876493

[7] Manicklal S Emery VC Lazzarotto T Boppana SB Gupta RK. The “silent” global burden of congenital cytomegalovirus. Clin Microbiol Rev. (2013) 26:86–102. 10.1128/CMR.00062-12 23297260 PMC3553672

[8] The American College of Obstetricians and Gynecologists Practice bulletin no. 151: cytomegalovirus, parvovirus B19, varicella zoster, and toxoplasmosis in pregnancy. Obstet Gynecol. (2015) 125:1510–25. 10.1097/01.AOG.0000466430.19823.53 26000539

[9] Rawlinson WD Boppana SB Fowler KB Kimberlin DW Lazzarotto T Alain S. Congenital cytomegalovirus infection in pregnancy and the neonate: consensus recommendations for prevention, diagnosis, and therapy. Lancet Infect Dis. (2017) 17:e177–88. 10.1016/S1473-3099(17)30143-3 28291720

[10] Cahill AG Odibo AO Stamilio DM Macones GA. Screening and treating for primary cytomegalovirus infection in pregnancy: where do we stand? A decision-analytic and economic analysis. Am J Obstet Gynecol. (2009) 201:466.e1–7. 10.1016/j.ajog.2009.07.056 19782961

[11] Revello MG Tibaldi C Masuelli G Frisina V Sacchi A Furione M. Prevention of primary cytomegalovirus infection in pregnancy. EBioMed. (2015) 2:1205–10. 10.1016/j.ebiom.2015.08.003 26501119 PMC4588434

[12] Naessens A Casteels A Decatte L Foulon W. A serologic strategy for detecting neonates at risk for congenital cytomegalovirus infection. J Pediatr. (2005) 146:194–7. 10.1016/j.jpeds.2004.09.025 15689906

[13] Lilleri D Gerna G. Maternal immune correlates of protection from human cytomegalovirus transmission to the fetus after primary infection in pregnancy. Rev Med Virol. (2017) 27:e1921. 10.1002/rmv.1921 28008685

[14] Guerra B Simonazzi G Banfi A Lazzarotto T Farina A Lanari M. Impact of diagnostic and confirmatory tests and prenatal counseling on the rate of pregnancy termination among women with positive cytomegalovirus immunoglobulin M antibody titers. Am J Obstet Gynecol. (2007) 196:221.e1–6. 10.1016/j.ajog.2006.08.039 17346528

[15] Wulff CB Gerds TA Rode L Ekelund CK Petersen OB Tabor A. Risk of fetal loss associated with invasive testing following combined first-trimester screening for Down syndrome: a national cohort of 147,987 singleton pregnancies. Ultrasound Obstet Gynecol. (2016) 47:38–44. 10.1002/uog.15820 26581188

[16] Prince HE Lape-Nixon M. Role of cytomegalovirus (CMV) IgG avidity testing in diagnosing primary CMV infection during pregnancy. Clin Vaccine Immunol. (2014) 21:1377–84. 10.1128/CVI.00487-14 25165026 PMC4266349

[17] Lazzarotto T Guerra B Gabrielli L Lanari M Landini MP. Update on the prevention, diagnosis and management of cytomegalovirus infection during pregnancy. Clin Microbiol Infect. (2011) 17:1285–93. 10.1111/j.1469-0691.2011.03564.x 21631642

[18] Faure-Bardon V Magny JF Parodi M Couderc S Garcia P Maillotte AM. Sequelae of congenital cytomegalovirus following maternal primary infections are limited to those acquired in the first trimester of pregnancy. Clin Infect Dis. (2019) 69:1526–32. 10.1093/cid/ciy1128 30596974

[19] Furione M Sarasini A Arossa A Fornara C Lilleri D Perez L. False human cytomegalovirus IgG-positivity at prenatal screening. J Clin Virol. (2018) 104:34–8. 10.1016/j.jcv.2018.04.009 29705613

[20] Berth M Grangeot-Keros L Heskia F Dugua JM Vauloup-Fellous C. Analytical issues possibly affecting the performance of commercial human cytomegalovirus IgG avidity assays. Eur J Clin Microbiol Infect Dis. (2014) 33:1579–84. 10.1007/s10096-014-2109-8 24781005

[21] Leruez-Ville M Magny JF Couderc S Pichon C Parodi M Bussieres L. Risk factors for congenital cytomegalovirus infection following primary and nonprimary maternal infection: a prospective neonatal screening study using polymerase chain reaction in saliva. Clin Infect Dis. (2017) 65:398–404. 10.1093/cid/cix337 28419213

[22] Puhakka L Lappalainen M Lonnqvist T Niemensivu R Lindahl P Nieminen T. The burden of congenital cytomegalovirus infection: a prospective cohort study of 20 000 infants in Finland. J Pediatric Infect Dis Soc. (2018) 8:205–12. 10.1093/jpids/piy027 29554325

[23] Wissel N Hanschmann K-M Scheiblauer H the Collaborative Study Group Report of the WHO Collaborative Study to Establish the First International Standard for Detection of IgG Antibodies to Cytomegalovirus (anti-CMV IgG). Available online at: http://www.who.int/biologicals/expert_committee/BS2322_WHO_IS_aCMV-IgG_ECBS_report_final_300617.pdf (accessed September 12, 2019).

[24] Maudry A Chene G Chatelain R Patural H Bellete B Tisseur B. Bicentric evaluation of six anti-toxoplasma immunoglobulin G (IgG) automated immunoassays and comparison to the Toxo II IgG Western blot. Clin Vaccine Immunol. (2009) 16:1322–6. 10.1128/CVI.00128-09 19587151 PMC2745007

[25] Vauloup-Fellous C. Standardization of rubella immunoassays. J Clin Virol. (2018) 102:34–8. 10.1016/j.jcv.2018.02.006 29486385

[26] Zavattoni M Furione M Lanzarini P Arossa A Rustico M Tassis B. Monitoring of human cytomegalovirus DNAemia during primary infection in transmitter and non-transmitter mothers. J Clin Virol. (2016) 82:89–93. 10.1016/j.jcv.2016.07.005 27467018

[27] Simonazzi G Cervi F Zavatta A Pellizzoni L Guerra B Mastroroberto M. Congenital cytomegalovirus infection: prognostic value of maternal DNAemia at amniocentesis. Clin Infect Dis. (2017) 64:207–10. 10.1093/cid/ciw700 27986666

[28] Delforge ML Costa E Brancart F Goldman D Montesinos I Zaytouni S. Presence of cytomegalovirus in urine and blood of pregnant women with primary infection might be associated with fetal infection. J Clin Virol. (2017) 90:14–17. 10.1016/j.jcv.2017.03.004 28319846

[29] Guerra B Simonazzi G Puccetti C Lanari M Farina A Lazzarotto T. Ultrasound prediction of symptomatic congenital cytomegalovirus infection. Am J Obstet Gynecol. (2008) 198:380.e1–7. 10.1016/j.ajog.2007.09.052 18191802

[30] Liesnard C Donner C Brancart F Gosselin F Delforge ML Rodesch F. Prenatal diagnosis of congenital cytomegalovirus infection: prospective study of 237 pregnancies at risk. Obstet Gynecol. (2000) 95(6 Pt 1):881–8. 10.1097/00006250-200006000-00019 10831985

[31] Enders M Daiminger A Exler S Ertan K Enders G Bald R. Prenatal diagnosis of congenital cytomegalovirus infection in 115 cases: a 5 years' single center experience. Prenat Diagn. (2017) 37:389–98. 10.1002/pd.5025 28207161

[32] Society for Maternal-Fetal Medicine (SMFM) Hughes BL Gyamfi-Bannerman C. Diagnosis and antenatal management of congenital cytomegalovirus infection. Am J Obstet Gynecol. (2016) 214:B5–11. 10.1016/j.ajog.2016.02.042 26902990

[33] Farkas N Hoffmann C Ben-Sira L Lev D Schweiger A Kidron D. Does normal fetal brain ultrasound predict normal neurodevelopmental outcome in congenital cytomegalovirus infection? Prenat Diagn. (2011) 31:360–6. 10.1002/pd.2694 21413035

[34] Faure-Bardon V Millischer AE Deloison B Sonigo P Grévent D Salomon L. Refining the prognosis of fetuses infected with cytomegalovirus in the first trimester of pregnancy by serial prenatal assessment: a single-centre retrospective study. BJOG. (2020) 127:355–62. 10.1111/1471-0528.15935 31505103

[35] Picone O Simon I Benachi A Brunelle F Sonigo P. Comparison between ultrasound and magnetic resonance imaging in assessment of fetal cytomegalovirus infection. Prenat Diagn. (2008) 28:753–8. 10.1002/pd.2037 18551722

[36] Lipitz S Yinon Y Malinger G Yagel S Levit L Hoffman C. Risk of cytomegalovirus-associated sequelae in relation to time of infection and findings on prenatal imaging. Ultrasound Obstet Gynecol. (2013) 41:508–14. 10.1002/uog.12377 23288698

[37] Leruez-Ville M Stirnemann J Sellier Y Guilleminot T Dejean A Magny JF. Feasibility of predicting the outcome of fetal infection with cytomegalovirus at the time of prenatal diagnosis. Am J Obstet Gynecol. (2016) 215:342.e1–9. 10.1016/j.ajog.2016.03.052 27063062

[38] Enders G Bader U Lindemann L Schalasta G Daiminger A. Prenatal diagnosis of congenital cytomegalovirus infection in 189 pregnancies with known outcome. Prenat Diagn. (2001) 21:362–77. 10.1002/pd.59 11360277

[39] Uematsu M Haginoya K Kikuchi A Hino-Fukuyo N Ishii K Shiihara T. Asymptomatic congenital cytomegalovirus infection with neurological sequelae: a retrospective study using umbilical cord. Brain Dev. (2016) 38:819–26. 10.1016/j.braindev.2016.03.006 27068877

[40] Vancor E Shapiro ED Loyal J. Results of a targeted screening program for congenital cytomegalovirus infection in infants who fail newborn hearing screening. J Pediatric Infect Dis Soc. (2019) 8:55–9. 10.1093/jpids/pix105 29373759 PMC6437837

[41] Diener ML Zick CD McVicar SB Boettger J Park AH. Outcomes from a hearing-targeted cytomegalovirus screening program. Pediatrics. (2017) 139:e20160789. 10.1542/peds.2016-0789 28119425

[42] Williams EJ Kadambari S Berrington JE Luck S Atkinson C Walter S. Feasibility and acceptability of targeted screening for congenital CMV-related hearing loss. Arch Dis Child Fetal Neonatal Ed. (2014) 99:F230–6. 10.1136/archdischild-2013-305276 24596404

[43] Beswick R David M Higashi H Thomas D Nourse C Koh G. Integration of congenital cytomegalovirus screening within a newborn hearing screening programme. J Paediatr Child Health. (2019) 55:1381–8. 10.1111/jpc.14428 30916438

[44] Courtmans I Mancilla V Ligny C Le Bon SD Naessens A Foulon I. Incidence of congenital CMV in children at a hearing rehabilitation center. B-ENT. (2015) 11:303–8. 26891544

[45] Cannon MJ Griffiths PD Aston V Rawlinson WD. Universal newborn screening for congenital CMV infection: what is the evidence of potential benefit? Rev Med Virol. (2014) 24:291–307. 10.1002/rmv.1790 24760655 PMC4494732

[46] Williams EJ Gray J Luck S Atkinson C Embleton ND Kadambari S. First estimates of the potential cost and cost saving of protecting childhood hearing from damage caused by congenital CMV infection. Arch Dis Child Fetal Neonatal Ed. (2015) 100:F501–6. 10.1136/archdischild-2014-306756 26122458

[47] Gantt S Dionne F Kozak FK Goshen O Goldfarb DM Park AH. Cost-effectiveness of universal and targeted newborn screening for congenital cytomegalovirus infection. JAMA Pediatr. (2016) 170:1173–80. 10.1001/jamapediatrics.2016.2016 27723885

[48] Fowler K Targeted CMV screening and hearing management of children with congenital cytomegalovirus infection. ENT Audiol News. (2018) 27.

[49] Fowler KB McCollister FP Sabo DL Shoup AG Owen KE Woodruff JL. A targeted approach for congenital cytomegalovirus screening within newborn hearing screening. Pediatrics. (2017) 139:e20162128. 10.1542/peds.2016-2128 28049114 PMC5260148

[50] Lanzieri TM Chung W Flores M Blum P Caviness AC Bialek SR. Hearing loss in children with asymptomatic congenital cytomegalovirus infection. Pediatrics. (2017) 139:e20162610. 10.1542/peds.2016-2610 28209771 PMC5330400

[51] Foulon I Vleurinck L Kerkhofs K Gordts F. Hearing configuration in children with cCMV infection and proposal of a flow chart for hearing evaluation. Int J Audiol. (2015) 54:714–9. 10.3109/14992027.2015.1046506 26068302

[52] Foulon I De Brucker Y Buyl R Lichtert E Verbruggen K Pierard D. Hearing loss with congenital cytomegalovirus infection. Pediatrics. (2019) 144:e20183095. 10.1542/peds.2018-3095 31266824

[53] Marsico C Kimberlin DW. Congenital cytomegalovirus infection: advances and challenges in diagnosis, prevention and treatment. Ital J Pediatr. (2017) 43:38. 10.1186/s13052-017-0358-8 28416012 PMC5393008

[54] de Vries JJ van der Eijk AA Wolthers KC Rusman LG Pas SD Molenkamp R. Real-time PCR versus viral culture on urine as a gold standard in the diagnosis of congenital cytomegalovirus infection. J Clin Virol. (2012) 53:167–70. 10.1016/j.jcv.2011.11.006 22177273

[55] Boppana SB Ross SA Shimamura M Palmer AL Ahmed A Michaels MG. Saliva polymerase-chain-reaction assay for cytomegalovirus screening in newborns. N Engl J Med. (2011) 364:2111–8. 10.1056/NEJMoa1006561 21631323 PMC3153859

[56] Lanzieri TM Dollard SC Josephson CD Schmid DS Bialek SR. Breast milk-acquired cytomegalovirus infection and disease in VLBW and premature infants. Pediatrics. (2013) 131:e1937–45. 10.1542/peds.2013-0076 23713111 PMC4850548

[57] Wang L Xu X Zhang H Qian J Zhu J. Dried blood spots PCR assays to screen congenital cytomegalovirus infection: a meta-analysis. Virol J. (2015) 12:60. 10.1186/s12985-015-0281-9 25889596 PMC4408583

[58] Atkinson C Emery VC Griffiths PD. Development of a novel single tube nested PCR for enhanced detection of cytomegalovirus DNA from dried blood spots. J Virol Methods. (2014) 196:40–4. 10.1016/j.jviromet.2013.10.029 24184085

[59] Kimberlin DW Acosta EP Sanchez PJ Sood S Agrawal V Homans J. Pharmacokinetic and pharmacodynamic assessment of oral valganciclovir in the treatment of symptomatic congenital cytomegalovirus disease. J Infect Dis. (2008) 197:836–45. 10.1086/528376 18279073

[60] Luck SE Emery VC Atkinson C Sharland M Griffiths PD. Compartmentalized dynamics of cytomegalovirus replication in treated congenital infection. J Clin Virol. (2016) 82:152–8. 10.1016/j.jcv.2016.07.018 27500364

[61] Walter S Atkinson C Sharland M Rice P Raglan E Emery VC. Congenital cytomegalovirus: association between dried blood spot viral load and hearing loss. Arch Dis Child Fetal Neonatal Ed. (2008) 93:F280–5. 10.1136/adc.2007.119230 18039747

[62] Working Group of Scientific Medical Societies Laboratory Diagnosis of Pregnancy-Relevant Viral Infections. Available online at: https://www.awmf.org/uploads/tx_szleitlinien/093-001l_S2k_Labordiagnostik_schwangerschaftsrelevanter_Virusinfektionen_2014-05-abgelaufen.pdf (accessed September 12, 2019).

[63] Nigro G Adler SP La Torre R Best AM. Passive immunization during pregnancy for congenital cytomegalovirus infection. N Engl J Med. (2005) 353:1350–62. 10.1056/NEJMoa043337 16192480

[64] Blázquez-Gamero D Galindo Izquierdo A Del Rosal T Baquero-Artigao F Izquierdo Méndez N Soriano-Ramos M. Prevention and treatment of fetal cytomegalovirus infection with cytomegalovirus hyperimmune globulin: a multicenter study in Madrid. J Matern Fetal Neonatal Med. (2019) 32:617–25. 10.1080/14767058.2017.1387890 28978246

[65] Kagan KO Enders M Schampera MS Baeumel E Hoopmann M Geipel A. Prevention of maternal-fetal transmission of cytomegalovirus after primary maternal infection in the first trimester by biweekly hyperimmunoglobulin administration. Ultrasound Obstet Gynecol. (2019) 53:383–9. 10.1002/uog.19164 29947159

[66] Revello MG Lazzarotto T Guerra B Spinillo A Ferrazzi E Kustermann A. A randomized trial of hyperimmune globulin to prevent congenital cytomegalovirus. N Engl J Med. (2014) 370:1316–26. 10.1056/NEJMoa1310214 24693891

[67] ClinicalTrials.gov. A Randomized Trial to Prevent Congenital Cytomegalovirus (CMV) (cCMV). Available online at: https://clinicaltrials.gov/ct2/show/NCT01376778 (accessed August 23, 2019).

[68] Kimberlin DW Jester PM Sanchez PJ Ahmed A Arav-Boger R Michaels MG. Valganciclovir for symptomatic congenital cytomegalovirus disease. N Engl J Med. (2015) 372:933–43. 10.1056/NEJMoa1404599 25738669 PMC4401811

[69] Kimberlin DW Lin CY Sanchez PJ Demmler GJ Dankner W Shelton M. Effect of ganciclovir therapy on hearing in symptomatic congenital cytomegalovirus disease involving the central nervous system: a randomized, controlled trial. J Pediatr. (2003) 143:16–25. 10.1016/S0022-3476(03)00192-6 12915819

[70] VALCYTE® Patient Information Available online at: https://www.gene.com/download/pdf/valcyte_patientinfo.pdf (accessed September 12, 2019).

[71] Cho JC Le AD Locke SC. Letermovir for prophylaxis of cytomegalovirus in allogeneic hematopoietic stem cell recipients. Drugs Today. (2018) 54:361–8. 10.1358/dot.2018.54.6.2833982 29998227

[72] Le J Zembillas A Stanton M Dahl E Hanna R Flagg A Letermovir for secondary cytomegalovirus (CMV) prophylaxis in a pediatric stem cell transplant patient. Biol Blood Marrow Transplant. (2019) 25:S282–3. 10.1016/j.bbmt.2018.12.354

[73] ClinicalTrials.gov. Randomized Controlled Trial of Valganciclovir for Cytomegalovirus Infected Hearing Impaired Infants (ValEAR). Available online at: https://clinicaltrials.gov/ct2/show/NCT03107871 (accessed September 12, 2019).

[74] ClinicalTrials.gov. Asymptomatic Congenital CMV Treatment. Available online at: https://clinicaltrials.gov/ct2/show/NCT03301415 (accessed September 12, 2019).

[75] ClinicalTrials.gov. Valganciclovir Therapy in Infants and Children With Congenital CMV Infection and Hearing Loss. Available online at: https://clinicaltrials.gov/ct2/show/NCT01649869 (accessed September 12, 2019).

[76] Schleiss MR Permar SR Plotkin SA. Progress toward development of a vaccine against congenital cytomegalovirus infection. Clin Vaccine Immunol. (2017) 24:e00268–17. 10.1128/CVI.00268-17 29046308 PMC5717185

[77] Krause PR Bialek SR Boppana SB Griffiths PD Laughlin CA Ljungman P. Priorities for CMV vaccine development. Vaccine. (2013) 32:4–10. 10.1016/j.vaccine.2013.09.042 24129123 PMC4623576

[78] N'Diaye DS Launay O Picone O Tsatsaris V Azria E Rozenberg F. Cost-effectiveness of vaccination against cytomegalovirus (CMV) in adolescent girls to prevent infections in pregnant women living in France. Vaccine. (2018) 36:1285–96. 10.1016/j.vaccine.2018.01.042 29397227

[79] Reichman O Miskin I Sharoni L Eldar-Geva T Goldberg D Tsafrir A. Preconception screening for cytomegalovirus: an effective preventive approach. Biomed Res Int. (2014) 2014:135416. 10.1155/2014/135416 25013756 PMC4075035

[80] de Vries JJ van Zwet EW Dekker FW Kroes AC Verkerk PH Vossen AC. The apparent paradox of maternal seropositivity as a risk factor for congenital cytomegalovirus infection: a population-based prediction model. Rev Med Virol. (2013) 23:241–9. 10.1002/rmv.1744 23559569

[81] ClinicalTrials.gov. Reducing Acquisition of CMV Through Antenatal Education (RACEFIT). Available online at: https://clinicaltrials.gov/ct2/show/NCT03511274 (accessed September 12, 2019).

[82] Retzler J Hex N Bartlett C Webb A Wood S Star C. Economic cost of congenital CMV in the UK. Arch Dis Child. (2019) 104:559–63. 10.1136/archdischild-2018-316010 30472664

